# Comparative clinical outcome following individualized follitropin delta dosing in Chinese women undergoing ovarian stimulation for in vitro fertilization /intracytoplasmic sperm injection

**DOI:** 10.1186/s12958-022-01016-y

**Published:** 2022-10-04

**Authors:** Rui Yang, Yunshan Zhang, Xiaoyan Liang, Xueru Song, Zhaolian Wei, Jianqiao Liu, Yezhou Yang, Jichun Tan, Qingxue Zhang, Yingpu Sun, Wei Wang, Weiping Qian, Lei Jin, Shuyu Wang, Yang Xu, Jing Yang, Marie Goethberg, Bernadette Mannaerts, Wen Wu, Zugeng Zheng, Jie Qiao

**Affiliations:** 1 Center for Reproductive Medicine, Department of Obstetrics and Gynecology, Peking University Third Hospital, Beijing, China; 2 Tianjin Central Hospital of Gynaecology and Obstetrics, Tianjin, China; 3 The Sixth Affiliated Hospital of Sun Yat-Sen University, Guangzhou, China; 4 Tianjin Medical University General Hospital, Tianjin, China; 5 The First Affiliated Hospital of An’hui Medical University, Hefei, China; 6 The Third Affiliated Hospital of Guangzhou Medical University, Guangzhou, China; 7 West China Second University Hospital, Sichuan University, Chengdu, Sichuan China; 8 ShengJing Hospital of China Medical University, Shenyang, China; 9 SunYat-Sen Memorial Hospital, Sun Yat-Sen University, Guangzhou, China; 10 The First Affiliated Hospital of Zhengzhou University, Zhengzhou, China; 11 Jiangsu Province Hospital, The First Affiliated Hospital With Nanjing Medical University, Nanjing, China; 12 Peking University Shenzhen Hospital, Shenzhen, China; 13 Tongji Hospital, Tongji Medical College of HUST, Tongji Medical College, Wuhan, China; 14 Beijing Obstetrics and Gynecology Hospital, Capital Medical University, Beijing, China; 15 Peking University First Hospital, Beijing, China; 16 Renmin Hospital of Wuhan University, Hubei General Hospital, Wuhan, China; 17 Global Biometrics, Ferring Pharmaceuticals, Copenhagen, Denmark; 18 Reproductive Medicine and Maternal Health, Ferring Pharmaceuticals, Copenhagen, Denmark; 19 Ferring Pharmaceuticals, Shanghai, China

**Keywords:** Follitropin delta, Algorithm, Individualized dosing, Ongoing pregnancy, Ovarian hyperstimulation syndrome, Dose equivalence

## Abstract

**Background:**

To compare the efficacy and safety of follitropin delta in its individualized fixed-dose regimen with follitropin alfa in a conventional adjustable dosing regimen in Chinese women.

**Methods:**

This was a subgroup analysis of the randomized, multi-center, assessor-blind, non-inferiority trial (GRAPE) including 759 Chinese women (aged 20–40 years) recruited in 16 reproductive medicine clinics in China. Women were randomized in a 1:1 ratio to be treated with either follitropin delta dose based on anti-Müllerian hormone (AMH) and body weight or conventional dosing with follitropin alfa following a gonadotropin-releasing hormone (GnRH) antagonist protocol. The primary outcome was ongoing pregnancy rate assessed 10–11 weeks after embryo transfer in the fresh cycle (non-inferiority margin -10.0%).

**Results:**

378 in the follitropin delta group and 381 in the follitropin alfa group were randomized and exposed. Non-inferiority was confirmed with respect to ongoing pregnancy with rates of 31.0% vs. 25.7% for follitropin delta compared to follitropin alfa, estimated mean difference of 5.1% (95% confidence interval (CI) -1.3% to 11.5%). The clinical pregnancy rate (35.4% vs. 31.5%, *P* = 0.239) and live birth rate (31.0% vs. 25.5%, *P* = 0.101) were comparable between the follitropin delta group and the follitropin alfa group. Overall, the individualized follitropin delta treatment resulted in fewer oocytes retrieved compared to follitropin alfa treatment (10.3 ± 6.2 vs. 12.5 ± 7.5, *P* < 0.001), which was mainly due to fewer oocytes (10.5 ± 6.4 vs. 13.9 ± 7.8) in women with AMH ≥ 15 pmol/L. Accordingly there was a lower incidence of early ovarian hyper-stimulation syndrome (OHSS) and/or preventive interventions (6.1% vs. 11.0%, *P* = 0.013). A daily follitropin delta dose of 10.2 µg (95% CI: 9.3—11.2 µg) was estimated to provide the same number of oocytes retrieved as a starting dose of 150 IU/d of follitropin alfa.

**Conclusion:**

Follitropin delta in its individualized fixed-dose regimen showed similar efficacy and improved safety compared with follitropin alfa in a conventional adjustable dosing regimen in Chinese women.

**Clinical trial registration number:**

NCT03296527.

**Supplementary Information:**

The online version contains supplementary material available at 10.1186/s12958-022-01016-y.

## Background

In modern assisted reproductive technology (ART), individualized treatment aims at an improved balance with respect to efficacy and safety outcome for each patient. Consistent with recent reports comparing individualized and conventional controlled ovarian stimulation (COS), most clinicians use ovarian reserve markers like AMH and/or antral follicle count (AFC) for decision-making to tailor the most optimal starting dose for each patient [1, 2]. Recently, an individualized dosing algorithm of follitropin delta applying serum AMH and body weight to determine the follitropin delta dose has been shown to decrease the incidence of moderate or severe ovarian hyperstimulation syndrome (OHSS) as well as incidence of preventive interventions for early OHSS compared with conventional treatment in women without compromising the live birth rates [1]. Especially the risk of OHSS following ovarian stimulation may prevent fresh embryo transfer, which increases treatment burden and time to pregnancy [3].

AMH, a paracrine factor implicated in the regulation of early follicular growth, is now widely been used as a quantitative marker of ovarian reserve to predict the response to ovarian stimulation [4, 5]. Meanwhile, the recent development of automated AMH assays improved the accuracy and consistency of AMH testing among different in vitro fertilization (IVF) centers [6, 7]. A study based on Chinese population clarified that the stature and body weight of female and male urban Han people positively correlated with latitude [8]. There is a huge latitude span of the territory in China, the mean body weight of women living in the north is higher than of women living in the south [8]. Since serum FSH levels are inversely related with body weight, women with a higher body weight have lower serum FSH exposure than women with a lower body weight [9]. Including body weight in the dosing algorithm takes into account patients’ distribution volume, which lowers the variability of FSH exposure [10, 11]. Inappropriate daily FSH dosing is known to result in a higher frequency of cycle cancellation due to poor response and transfer cancellations due to excessive response. Therefore, the optimal FSH dose should be considered for each patient, taking into account the ovarian reserve and other relevant characteristics, thus preventing cycle cancellation.

Follitropin delta is a novel recombinant FSH (rFSH) preparation developed with an individualized dosing regimen based on each patient’s serum AMH concentration and body weight [1, 12]. This individualized dosing regimen has been used in three randomized, controlled, multi-center phase III trials [1, 13, 14]. ESTHER-1/2 [1, 15] was conducted in European countries, Canada and Brazil, and showed that the follitropin delta dose was significantly reduced compared to follitropin alfa with non-inferior ongoing pregnancy rates and improved safety. After repeated cycles and cryopreserved cycles, the cumulative live birth rate assessed 4 weeks after birth were 60.3% in follitropin delta and 60.7% in follitropin alfa [15]. A trial conducted in Japan [13] showed non-inferiority with respect to oocytes retrieved and significantly fewer OHSS cases vs follitropin beta. The GRAPE trial [14] was conducted in Asia and included women from mainland China, South Korea, Vietnam and Taiwan. Similar to the ESTHER-1 trial [1], the FSH dose was significantly reduced compared to follitropin alfa with non-inferior ongoing pregnancy rates and improved safety [14].

This study is the subgroup analysis of GRAPE trial [14] aimed to compare the efficacy and safety outcomes of follitropin delta in its novel individualized algorithm-based dosing regimen to conventional dosing of follitropin alfa in Chinese women undergoing their first ART cycle, and to describe the effect of individualized dosing in women with AMH below 15 pmol/L and in women with AMH of 15 pmol/L or above.

## Methods

### Study design

A randomized, controlled, assessor-blind, parallel groups, multi-center, phase III, non-inferiority trial comparing follitropin delta in its individualized fixed-dose regimen with follitropin alfa in a conventional adjustable dose regimen was conducted at 1009 Asian patients in mainland China, South Korea, Taiwan and Vietnam (number NCT03296527). A detailed description of the elements of the trial design was described previously [14]. This was a subgroup analysis based on 759 patients (75.2%) recruited in mainland China. All women provided written, informed consent.

### Study participants

Chinese pre-menopausal women, aged 20–40 years, with a BMI between 17.5 and 32.0 kg/m^2^ and undergoing their first ovarian stimulation cycle for IVF/ intracytoplasmic sperm injection (ICSI), diagnosed with tubal infertility, unexplained infertility, endometriosis stage I/II or with partners diagnosed with male factor infertility were eligible for the study. The main exclusion criteria were women with endometriosis stage III/IV, history of recurrent miscarriage and with one or more follicles ≥ 10 mm observed before randomization. The full characterization of inclusion and exclusion criteria has been provided as supplementary table in published study (GRAPE trial) [14].

### Study procedures

Women were randomized in a 1:1 ratio to follitropin delta (Rekovelle, Ferring Pharmaceuticals) or follitropin alfa (Gonal-f; Merck Serono, Geneva, Switzerland) (Additional Fig. 1). The individualized follitropin delta (72 μg/2.16 ml) dose was determined by their serum AMH level at screening and body weight at randomization (AMH < 15 pmol/L, 12 μg/d; AMH ≥ 15 pmol/L, 0.19–0.10 μg/kg/d, minimum 6 μg/d and maximum 12 μg/d). Women randomized to follitropin alfa (900 IU/1.5 ml) started with a dose of 150 IU (11 µg) [16, 17] for the first 5 stimulation days, thereafter, the dose was adjusted by ± 75 IU based on individual response, with a maximum daily dose of 450 IU. FSH was initiated on days 2–3 of the menstrual cycle and a gonadotropin-releasing hormone (GnRH) antagonist (cetrorelix acetate, Cetrotide; Merck Serono, Geneva, Switzerland) 0.25 mg/d was initiated on day 6 and continued throughout the simulation period.

**Fig. 1:**
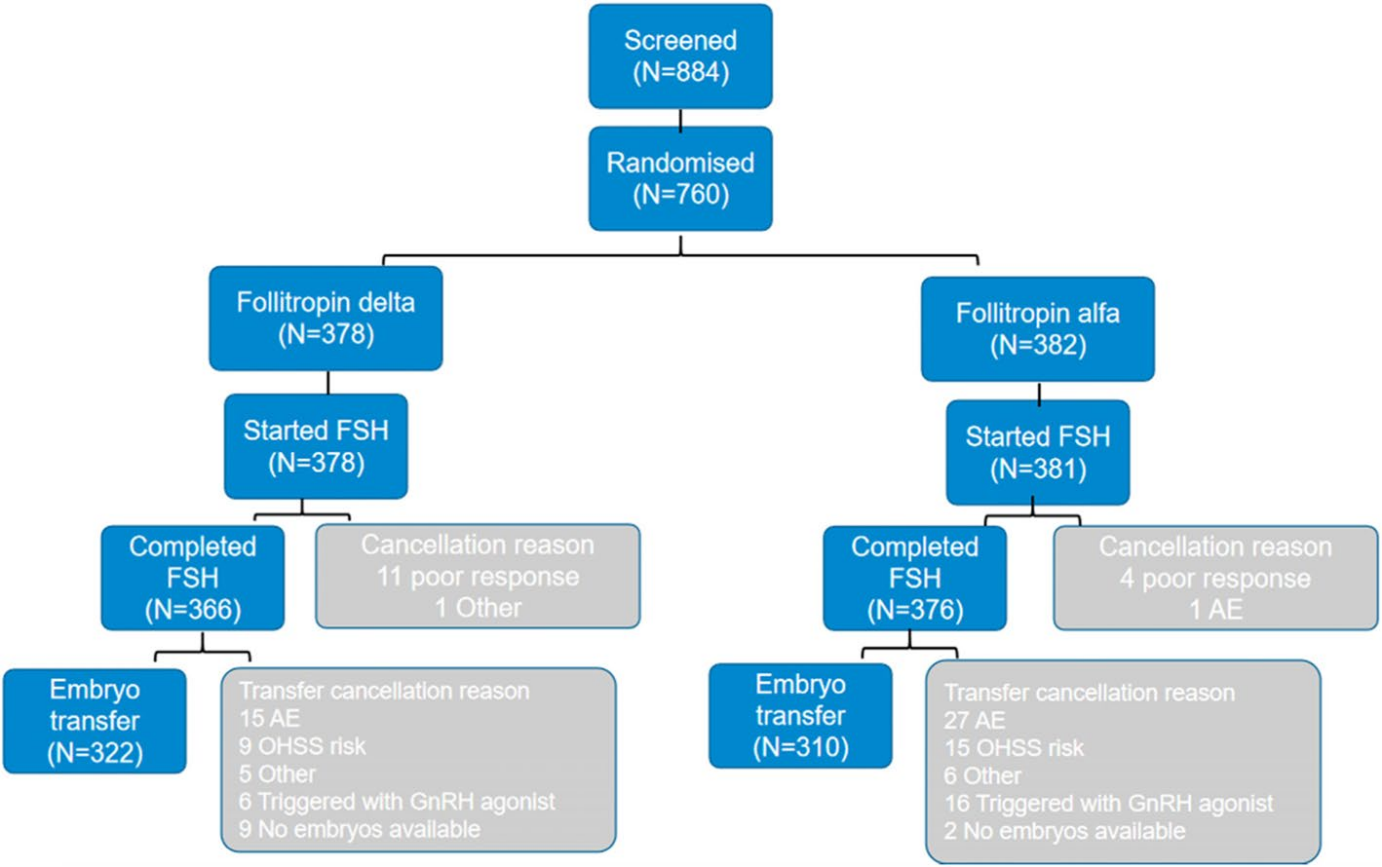
Flow chart showing the number of women at each stage of the clinical trial. Note: AMH, anti-Müllerian hormone; GnRH, gonadotropin-releasing hormone; hCG: human chorionic gonadotrophin

Details of the study procedure including human chorionic gonadotrophin (hCG) triggering, oocyte retrieval, embryo transfer, vaginal progesterone gel administration, timeline of βhCG test, clinical pregnancy, ongoing pregnancy, live-birth follow-up have been published previously [14].

### Study outcomes

The delivery of a healthy baby was the ultimate treatment success in this trial. The primary endpoint was ongoing pregnancy (defined as at least one intrauterine viable fetus 10–11 weeks after transfer). Secondary outcomes included other pregnancy outcomes, daily/total gonadotropin dose and duration of stimulation, oocytes and embryo development, and safety outcomes.

Other pregnancy outcomes included positive βhCG (defined as serum βhCG qualitative test performed 13–15 days after transfer), clinical pregnancy (defined as at least one gestational sac 5–6 weeks after transfer), vital pregnancy (defined as at least one intrauterine gestational sac with fetal heart 5–6 weeks after transfer), live birth (the birth of at least one live neonate) and live birth at 4 weeks (the presence of at least one live neonate 4 weeks after birth). The targeted ovarian response was defined as 8–14 oocytes retrieved.

Of the safety parameters, OHSS was an adverse event of special interest during controlled ovarian stimulation. Investigators recorded OHSS symptoms and used Golan’s classification system (1989) [18] to grade each OHSS case as mild (grade 1 and 2), moderate (grade 3) or as severe (grade 4 or 5). The criteria of OHSS were identical to those described previously by Qiao et al. [14] and in each detail by Višnová et al. [17].

Safety outcomes included adverse events such as early OHSS with onset ≤ 9 days after triggering of final follicular maturation and, preventive interventions for early OHSS defined as cycle cancellation due to excessive ovarian response or triggering of final follicular maturation with GnRH agonist with a single dose of 0.2 mg triptorelin.

In addition to the overall comparison between follitropin delta and follitropin alfa, the two treatment strategies were also compared within the two subgroups defined by AMH (< 15 pmol/l or ≥ 15 pmol/l).

Estimation of the dose equivalence factor of follitropin delta (ug) to follitropin alfa (IU) in terms of ovarian stimulation response was conducted post-hoc and compared to previously published data [16].

### Statistical analysis

All analyses are based on the subgroup of women recruited in China. The mean difference in ongoing pregnancy rates (follitropin delta—follitropin alfa) was estimated using the Mantel–Haenszel method, combining risk differences across age strata, and the 95% confidence interval (CI) calculated for the full analysis set. The non-inferiority margin was -10.0%. The Mantel–Haenszel method was also used to compare the two treatment groups on positive βhCG, clinical pregnancy, vital pregnancy and live birth rates.

The FSH dose (average daily dose and total dose), duration of stimulation, number of oocytes retrieved, fertilization rate, the number and quality of embryos on day 3 were compared between the follitropin delta group and the follitropin alfa group using the van Elteren test adjusted for AMH group (< 15 pmol/l or ≥ 15 pmol/l) and using the Wilcoxon’s test to compare treatments within each AMH group. A logistic regression model with treatment and AMH group as fixed factors was used to compare proportions of women that meet the targeted response (8–14 oocytes), excessive response and early OHSS and/or preventive interventions between treatment groups. The difference between follitropin delta and follitropin alfa was reported as an odds ratio (OR) including associated 95% CI and P-value based on the likelihood ratio test. In addition, the Fisher’s exact test was provided for the treatment comparisons within AMH group.

Analyses were made post-hoc to estimate the dose equivalence factor for the follitropin delta μg dose corresponding to 150 IU of follitropin alfa for endpoints related to ovarian response. Ovarian response parameters included number of oocytes retrieved, number of follicles with a diameter of 12 mm or more at the end of stimulation, and serum concentrations of oestradiol at the end of stimulation. The individualized follitropin delta dose was calculated for all women and the relationship between the follitropin delta dose and the ovarian response was approximated with a linear function (log-dose versus response) [16] for each treatment group. The point where the two regression lines intersect was the follitropin delta dose estimated to correspond to a starting dose of 150 IU/day follitropin alfa. The intersection point was estimated from the model parameters and the 95% CI for the estimate was derived and transformed to the linear scale using the delta method. The linear approximations were illustrated in figures including estimated means and 95% CI for each dose and the dose equivalence factor.

All statistical analyses were performed in SAS (SAS Institute Inc, version 9.4, Cary, NC, USA). A level of *P* < 0.05 was considered as statistically significant.

## Results

### Baseline characteristics

A total of 884 women were screened for eligibility and 760 (86.0%) women were randomized, of whom 759 were exposed to study drug: 378 to individualized follitropin delta (the follitropin delta group) and 381 to conventional follitropin alfa (the follitropin alfa group) (Fig. 1).

**Fig. 2:**
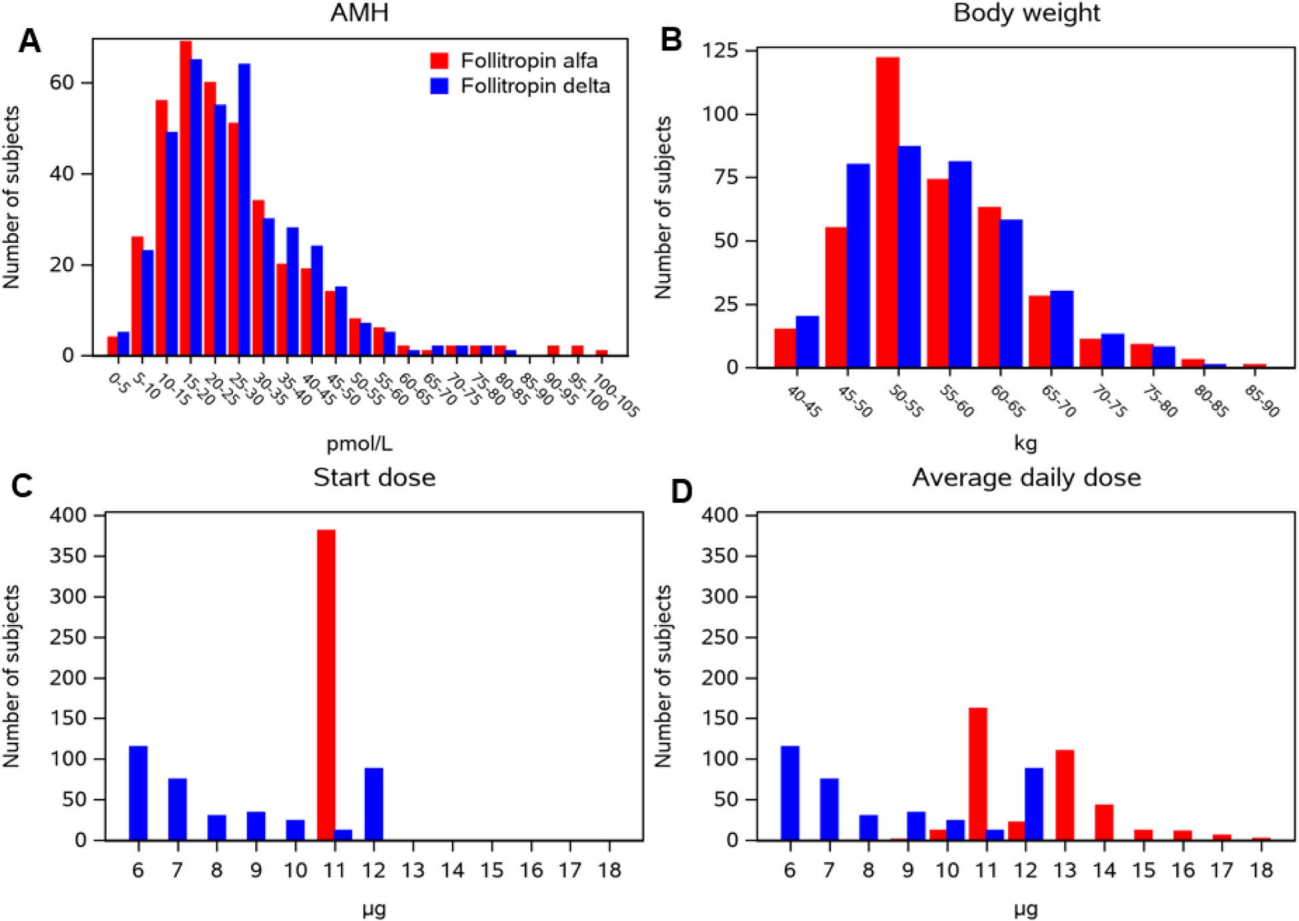
Adjustments of treatment doses according to AMH and body weight. Note: AMH, anti-Müllerian hormone.^a^Women randomized to follitropin delta were administered a fixed daily dose according to patients’ serum AMH concentration at screening and body weight at randomization (AMH < 15 pmol/L: 12 mg; AMH ≥ 15 pmol/L: 0.10–0.19 mg/kg, the maximum daily dose was 12 μg). ^b^Women randomized to follitropin alfa were administered a conventional daily dose of 150 IU (11 μg) for the first 5 days, thereafter the dose was adjusted up or down according to follicular response, the maximum daily dose was 450 IU

The mean (± standard deviation (SD)) age was similar in the follitropin delta group (30.6 ± 3.6 years) and in the follitropin alfa group (30.8 ± 3.6 years). The body weight and the AMH distributions were similar (Fig. 2A and B) with mean body weight (56.0 ± 8.0 kg vs. 56.5 ± 7.9 kg) and mean AMH (25.9 ± 13.6 pmol/l vs. 26.1 ± 16.1 pmol/l) respectively. The calculated starting dose using the follitropin delta algorithm based on the serum AMH concentration at screening and body weight at randomization, facilitates the simultaneous comparison of AMH and body weight between the two treatment groups. The mean algorithmic doses were 8.5 ± 2.3 μg/day vs. 8.8 ± 2.4 μg/day, respectively. The proportion of participants with AMH ≥ 15 pmol/l was 79.6% (301/378) for the follitropin delta group and 77.4% (295/381) for the follitropin alfa group. Treatment groups were also generally balanced with respect to other demographics and baseline characteristics (Table 1).

**Table 1:** Demographics and baseline characteristics of study population

Characteristics	Follitropin delta group (*n* = 378)	Follitropin alfa group (*n* = 381)
**Age** (years)	30.6 ± 3.6	30.8 ± 3.6
< 35	312 (82.5%)	313 (82.2%)
35–37	55 (14.6%)	52 (13.6%)
38–40	11 (2.9%)	16 (4.2%)
**Body weight** (kg)	56.0 ± 8.0	56.5 ± 7.9
**BMI** (kg/m^2^)	21.8 ± 2.7	22.0 ± 2.8
**Infertility history**		
Duration of infertility (months)	42.2 ± 24.4	45.3 ± 28.2
Primary infertility	244 (64.6%)	223 (58.5%)
**Reason for infertility**		
Unexplained infertility	74 (19.6%)	66 (17.3%)
Tubal infertility	197 (52.1%)	204 (53.5%)
Male factor	98 (25.9%)	106 (27.8%)
Endometriosis stage I/II	5 (1.3%)	2 (0.5%)
Other	4 (1.1%)	3 (0.8%)
**Endometrial thickness** (mm)	6.1 ± 2.2	6.1 ± 2.1
**Ovarian volume** (cm^3^)	5.0 ± 2.2	4.9 ± 2.5
**AFC**^a^	15.4 ± 6.2	15.0 ± 5.6
**Endocrine profile**^b^		
AMH (pmol/l)^c^	25.9 ± 13.6	26.1 ± 16.1
AMH < 15 pmol/l	77 (20.4%)	86 (22.6%)
AMH ≥ 15 pmol/l	301 (79.6%)	295 (77.4%)
FSH (IU/l)	7.3 (6.1–8.5)	7.2 (6.2–8.2)
LH (IU/l)	3.7 (2.9–4.8)	3.7 (2.8–4.9)
Estradiol (pmol/l)	140 (113.1–164.5)	142.2 (112.1–176.4)
Progesterone (nmol/L)	0.8 (0.8–2.1)	0.8 (0.8–2.1)
Inhibin A (ng/L)	5.0 (4.0–6.5)	5.3 (4.0–6.5)
Inhibin B (ng/L)	81.0 (62.0–101.5)	82.0 (67.0–101.0)
**Algorithmic dose (µg/day)**^d^	8.5 ± 2.3	8.8 ± 2.4

Note: Values are means ± SD, median (25%-75% percentiles) or n (%), unless otherwise stated. Data are for all patients unless otherwise stated

*AFC* Antral follicle count, *AMH* Anti-Müllerian hormone, *BMI* Body mass index, *FSH* Follicle-stimulating hormone, *LH* Luteinising hormone, *n* number of patients with observations, *SD* Standard deviation

^a^This measurement reports the total number of antral follicles with a diameter of ≥ 2 mm for both ovaries combined, assessed by transvaginal ultrasound at the day of starting ovarian stimulation

^b^The AMH values are based on the screening samples, while the remining endocrine parameters are based on the samples taken on stimulation day 1 before start of stimulation

^c^The serum concentration of AMH was assessed by a central laboratory using the Elecsys® AMH Plus immunoassay from Roche Diagnostics

^d^The algorithmic dose is the calculated using the follitropin delta algorithm based on the serum AMH concentration at screening and the body weight at randomization. The µg dose is rounded to the nearest 1/3 µg and applying a minimum dose of 6 µg and a maximum dose of 12 µg

### Pregnancy and live birth outcomes

Non-inferiority of individualized follitropin delta to conventional follitropin alfa was confirmed among women recruited from mainland China in respect to ongoing pregnancy since the lower bounds of the 95% CI were well above the pre-specified non-inferiority limit of -10.0%. The ongoing pregnancy rate was 31.0% (117/378) for the follitropin delta group versus 25.7% (98/381) for the follitropin alfa group, with a mean difference of 5.1% (95% CI: -1.3%; 11.5%).

**Fig. 3:**
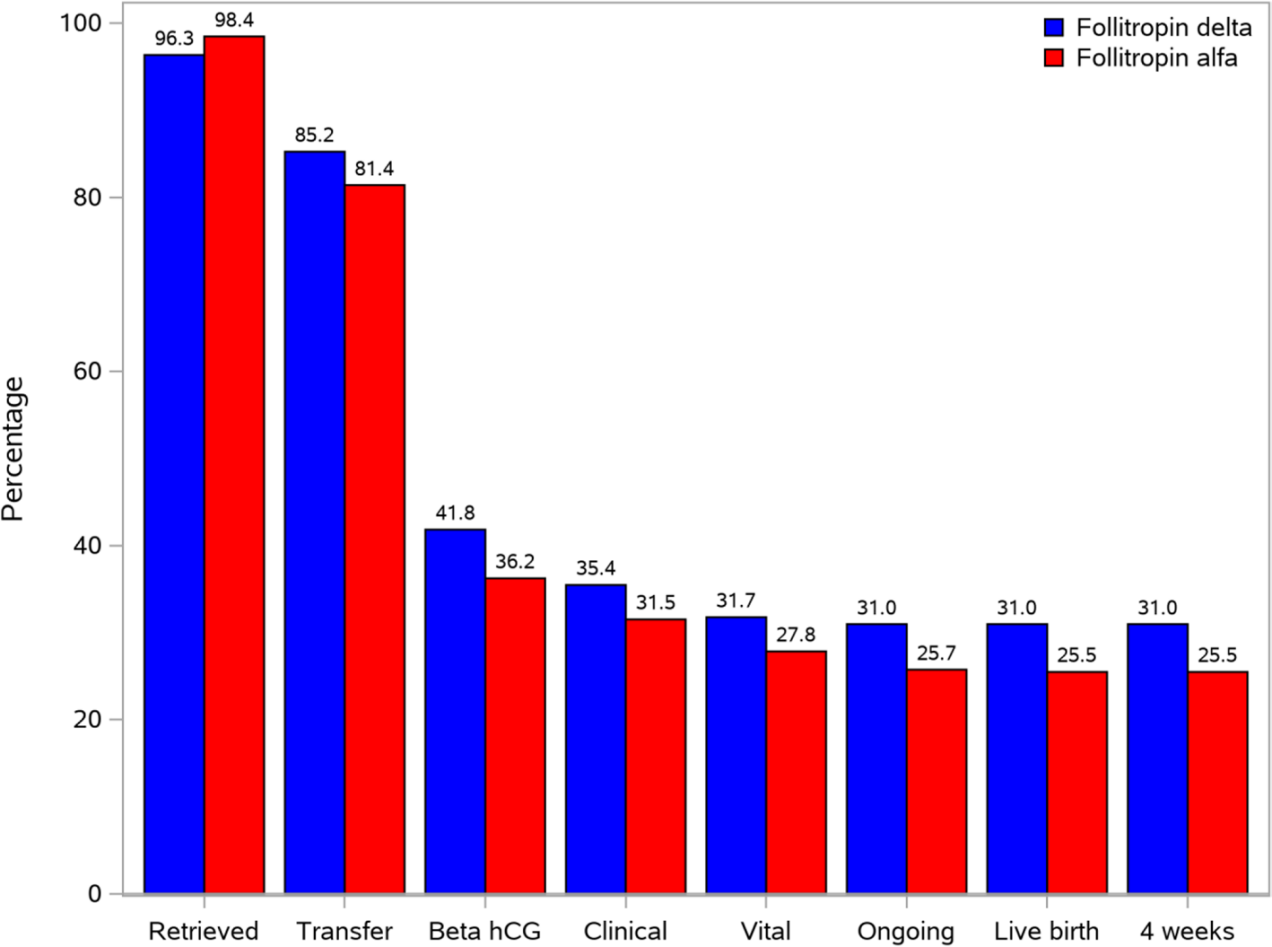
Pregnancy and live birth outcomes. Note: hCG: human chorionic gonadotrophin.^a^Rates are calculated per treatment group as the percentage of women in the FAS. The retrieved rate was defined as women with at least one oocyte retrieved. ^b^The transfer rate was defined as percentage of women with embryo transfer. ^c^βhCG rate was defined as percentage of women with a positive test of serum βhCG 2 weeks after transfer. ^d^Clinical pregnancy rate was defined as percentage of women with at least one gestational sac 5–6 weeks after transfer. ^e^Vital pregnancy rate was defined as percentage of women with at least one intrauterine gestational sac with fetal heart after transfer. ^f^Ongoing pregnancy rate was defined as percentage of women with at least one intrauterine viable fetus 10–11 weeks after transfer. ^g^Live birth rate was defined as percentage of women with birth of at least one live-born neonate. ^h^Live birth rate at 4 weeks after birth was defined as percentage of women with at least one live neonate 4 weeks after birth

No significant differences were seen for positive βhCG rate (41.8% [158/378] vs. 36.2% [138/381]), clinical pregnancy rate (35.4% [134/378] vs. 31.5% [120/381]), vital pregnancy rate (31.7% [120/378] vs. 27.8% [106/381]) and live birth rate (31.0% [117/378] vs. 25.5% [97/381]) in the follitropin delta group compared with the follitropin alfa group. There were no neonatal deaths in the period from birth to 4 weeks after birth (Fig. 3), hence the live rate at 4 weeks equals the live birth rates.

### Daily/total dose of rFSH and duration of stimulation

FSH stimulation was completed by 366 (96.8%) and 376 (98.7%) women in the follitropin delta and follitropin alfa group respectively, with cycle cancellations in 12 and 5 women in each group (Table 2).

**Table 2:** Efficacy and safety in the Chinese population

Variable	Follitropin delta (*n* = 378)	Follitropin alfa (*n* = 381)	*P*-value
**Gonadotropin use**			
Duration of stimulation (days)	9.2 ± 1.8	8.8 ± 1.5	0.025^a^
Total dose (μg)	76.7 ± 23.8	110.4 ± 31.2	< 0.001^a^
Average daily dose (μg/day)	8.5 ± 2.3	12.4 ± 1.6	< 0.001^a^
**Number of follicles at end of stimulation**			
Total number of follicles	12.8 ± 5.6	14.2 ± 6.5	0.005^a^
Follicles ≥ 12 mm	10.7 ± 5.0	12.0 ± 5.7	0.006^a^
Follicles ≥ 17 mm	4.3 ± 1.8	4.5 ± 2.0	0.359^a^
**Endocrine profile at end of stimulation**			
FSH (IU/l)	11.5	12.4	< 0.001^b^
LH (IU/l)	1.7	1.9	0.011^b^
Estradiol (pmol/l)	7001	9130	< 0.001^b^
Progesterone (nmol/l)	2.3	3.1	< 0.001^b^
Progesterone > 3.18 nmol/L	121 (32.4%)	190 (50.7%)	< 0.001^c^
Inhibin A (ng/l)	361.2	449.3	< 0.001^b^
Inhibin B (ng/l)	1022.6	1134.7	0.002^b^
**Cancellations**			
Cycle cancellation	12 (3.2%)	5 (1.3%)	0.085^c^
Cycle or transfer cancellation	56 (14.8%)	71 (18.6%)	0.126^c^
**Oocytes and embryos**			
Women with at least one oocyte retrieved	364 (96.3%)	375 (98.4%)	0.068^c^
Oocytes retrieved (n)	10.3 ± 6.2	12.5 ± 7.5	< 0.001^a^
Fertilized oocytes (n)	6.4 ± 4.2	7.7 ± 5.0	< 0.001^a^
Fertilization rate^d^ (%)	63.2 ± 22.8	62.8 ± 21.5	0.365^a^
Day 3 embryos (n)	7.3 ± 4.7	8.8 ± 5.5	< 0.001^a^
Day 3 good-quality embryos (n)	4.4 ± 3.8	5.3 ± 4.2	0.002^a^
**OHSS**			
Any OHSS	34 (9.0%)	36 (9.4%)	0.764^c^
Early OHSS (any grade)^e^	18 (4.8%)	28 (7.3%)	0.115^c^
Late OHSS (any grade)^f^	16 (4.2%)	8 (2.1%)	0.098^c^
Preventive interventions^g^ for early OHSS	6 (1.6%)	16 (4.2%)	0.025^c^
Early OHSS and/or preventive interventions	23 (6.1%)	42 (11.0%)	0.011^c^

Note: Values are means, mean ± SD, or n (%), unless otherwise stated

*AMH* Anti-Müllerian hormone, number of patients with observations, *FSH* Follicle-stimulating hormone, *LH* Luteinising hormone, *OHSS* Ovarian hyperstimulation syndrome, *SD* Standard deviation

^a^Van Elteren test adjusted for AMH group

^b^Multiplicative ANCOVA with treatment and AMH group as factors and the log transformed day 1 level as covariate

^c^Logistic regression model with treatment and AMH group as factors

^d^In women with at least one oocyte retrieved

^e^Onset ≤ 9 days after triggering of final follicular maturation

^f^Onset ≥ 10 days after triggering of final follicular maturation

^g^Preventive interventions included cycle cancellation due to excessive ovarian response and triggering of final follicular maturation with GnRH agonist

The average daily dose (mean ± SD) (8.5 ± 2.3 μg/day vs. 12.4 ± 1.6 μg/day) and the total dose (76.7 ± 23.8 μg vs. 110.4 ± 31.2 μg) were lower with follitropin delta than those with follitropin alfa (Table 2). The mean stimulation period was 0.5 day longer in the follitropin delta group compared to the follitropin alfa group (9.2 ± 1.8 days vs. 8.8 ± 1.5 days). Among women with AMH < 15 pmol/L, the starting dose of 12.0 μg was fixed throughout stimulation in the follitropin delta treatment group, whereas in the follotropin alfa treatment group the starting dose of 11 μg was adjusted to an average daily dose of 12.6 ± 1.7 μg (Fig. 2C and D). The total dose and the stimulation period were similar with mean 105.8 ± 16.7 μg vs. 110.2 ± 33.5 μg and 8.8 ± 1.4 days vs. 8.6 ± 1.6 days, respectively (Table 3). In women with AMH ≥ 15 pmol/L, the average daily dose (7.5 ± 1.7 μg/day vs. 12.3 ± 1.6 μg/day) and the average total dose (69.2 ± 19.2 μg vs. 110.4 ± 30.6 μg) were lower with follitropin delta than those with follitropin alfa. The stimulation period was (9.2 ± 1.9 days vs. 8.9 ± 1.5 days).

**Table 3:** Efficacy and safety in the Chinese population by AMH group

**Variable**	**AMH < 15 pmol/L**			**AMH** ≥ **15 pmol/L**		
	**Follitropin delta (*n* = 77)**	**Follitropin alfa (*n* = 86)**	***P*-value**	**Follitropin delta (*n* = 301)**	**Follitropin alfa (*n* = 295)**	***P*-value**
**Gonadotropin use**						
Duration of stimulation (days)	8.8 ± 1.4	8.6 ± 1.6	0.309^a^	9.2 ± 1.9	8.9 ± 1.5	0.047^a^
Total dose (μg)	105.8 ± 16.7	110.2 ± 33.5	0.568^a^	69.2 ± 19.2	110.4 ± 30.6	< 0.001^a^
Average daily dose (μg/day)	12.0 ± 0.0	12.6 ± 1.7	0.279^a^	7.5 ± 1.7	12.3 ± 1.6	< 0.001^a^
**Number of follicles at end of stimulation**						
Total number of follicles	10.3 ± 4.3	9.6 ± 4.4	0.302^a^	13.4 ± 5.8	15.6 ± 6.4	< 0.001^a^
Follicles ≥ 12 mm	8.8 ± 4.0	8.1 ± 4.1	0.238^a^	11.2 ± 5.2	13.1 ± 5.6	< 0.001^a^
Follicles ≥ 17 mm	4.0 ± 1.4	3.8 ± 1.3	0.309^a^	4.3 ± 1.9	4.7 ± 2.1	0.119^a^
**Endocrine profile at end of stimulation**						
FSH (IU/l)	17.0	13.3	< 0.001^b^	10.4	12.1	< 0.001^b^
LH (IU/l)	2.0	2.3	0.124^b^	1.6	1.8	0.036^b^
Estradiol (pmol/l)	6898	6239	0.179^b^	7029	10,204	< 0.001^b^
Progesterone (nmol/l)	3.1	2.7	0.215^b^	2.2	3.2	< 0.001^b^
Progesterone > 3.18 nmol/L	42 (54.5%)	39 (45.9%)	0.345^c^	79 (26.6%)	151 (52.1%)	< 0.001^c^
Inhibin A (ng/l)	341.3	310.2	0.159^b^	366.6	500.8	< 0.001^b^
Inhibin B (ng/l)	654.3	658.0	0.788^b^	1153.0	1320.2	< 0.001^b^
**Cancellations**						
Cycle cancellation	0 (0.0%)	1 (1.2%)	1.000^c^	12 (4.0%)	4 (1.4%)	0.073^c^
Cycle or transfer cancellation	6 (7.8%)	5 (5.8%)	0.757^c^	50 (16.6%)	66 (22.4%)	0.079^c^
**Oocytes and embryos**						
Women with at least one oocyte retrieved	76 (98.7%)	85 (98.8%)	1.000^c^	288 (95.7%)	290 (98.3%)	0.092^c^
Oocytes retrieved (n)	9.2 ± 4.9	7.7 ± 3.6	0.068^a^	10.5 ± 6.4	13.9 ± 7.8	< 0.001^b^
Fertilized oocytes (n)	6.0 ± 3.3	5.0 ± 2.9	0.057^a^	6.4 ± 4.5	8.5 ± 5.2	< 0.001^a^
Fertilization rate^d^ (%)	66.0 ± 21.6	66.3 ± 22.9	0.914^a^	62.5 ± 23.2	61.8 ± 21.1	0.280^a^
Day 3 embryos (n)	6.9 ± 3.8	5.7 ± 3.0	0.070^a^	7.4 ± 4.9	9.7 ± 5.7	< 0.001^a^
Day 3 good-quality embryos (n)	3.6 ± 2.9	3.4 ± 2.3	0.800^a^	4.6 ± 3.9	5.9 ± 4.5	< 0.001^a^
**OHSS**						
Any OHSS	4 (5.2%)	1 (1.2%)	0.190^c^	30 (10.0%)	35 (11.9%)	0.512^c^
Early OHSS (any grade)^e^	3 (3.9%)	0 (0.0%)	0.103^c^	15 (5.0%)	28 (9.5%)	0.039^c^
Late OHSS (any grade)^f^	1 (1.3%)	1 (1.2%)	1.000^c^	15 (5.0%)	7 (2.4%)	0.127^c^
Preventive interventions^g^ for early OHSS	0 (0.0%)	1 (1.2%)	1.000^c^	6 (2.0%)	15 (5.1%)	0.047^c^
Early OHSS and/or preventive interventions	3 (3.9%)	1 (1.2%)	0.345^c^	20 (6.6%)	41 (13.9%)	0.004^c^

Note: Values are means, mean ± SD, or n (%), unless otherwise stated

*AMH* Anti-Müllerian hormone, number of patients with observations, *FSH* Follicle-stimulating hormone, *LH* Luteinising hormone, *OHSS* Ovarian hyperstimulation syndrome, *SD* Standard deviation

^a^Wilcoxon test

^b^Multiplicative ANCOVA with treatment as factor and the log transformed day 1 level as covariate

^c^Fishers exact test

^d^In women with at least one oocyte retrieved

^e^Onset ≤ 9 days after triggering of final follicular maturation

^f^Onset ≥ 10 days after triggering of final follicular maturation

^g^Preventive interventions included cycle cancellation due to excessive ovarian response and triggering of final follicular maturation with GnRH agonist

### Follicular development and endocrine response

The total number of follicles ≥ 10 mm (12.8 ± 5.6 vs. 14.2 ± 6.5) and number of follicles ≥ 12 mm (10.7 ± 5.0 vs. 12.0 ± 5.7) at the end of simulation were both lower in the follitropin delta compared to the follitropin alfa group (Table 2). Likewise, the similar results were gained in women with AMH ≥ 15 pmol/l in terms of total number of follicles ≥ 10 mm (13.4 ± 5.8 vs. 15.6 ± 6.4) and number of follicles ≥ 12 mm (11.2 ± 5.2 vs. 13.1 ± 5.6) whereas, in women with AMH ≥ 15 pmol/l the treatment groups were similar (Table 3). There were no significant differences of total number of follicles ≥ 17 mm between two treatment groups.

At the end of stimulation, significantly (all *P* < 0.05) lower luteinizing hormone (LH), estradiol, progesterone, inhibin A and inhibin B were observed in the follitropin delta group than that in the follitropin alfa group (Table 2). The difference in endocrine profile was also noted in women with AMH ≥ 15 pmol/l, whereas the endocrine profile in women with AMH < 15 pmol/L were similar for the two treatments (Table 3). At the end of stimulation, mean serum-FSH reduced by 7% in the follitropin delta group compared to follitropin alfa, the corresponding reduction in women with AMH ≥ 15 pmol/L was 15%. In women with AMH ≥ 15 pmol/L the mean serum-FSH concentration was increased by 25% in the follitropin delta group compared to follitropin alfa. Since the FSH dose was similar in the two groups, this is explained by the longer half-life of follitropin delta.

### Oocytes and embryo development

A total of 364 women (96.3%) in the follitropin delta group and 375 women (98.4%) in the follitropin alfa group had at least one oocyte retrieved (Table 2). The mean number of oocytes retrieved was lower in the follitropin delta group compared to the follitropin alfa group (10.3 ± 6.2 vs. 12.5 ± 7.5). Among women with AMH < 15 pmol/L at screening, there were no significant differences between treatment groups in terms of number of oocytes retrieved (9.2 ± 4.9 vs 7.7 ± 3.6) (Table 3). Nevertheless, among women with AMH ≥ 15 pmol/l, follitropin delta was associated with fewer oocytes (10.5 ± 6.4 vs. 13.9 ± 7.8) as well as fewer fertilized oocytes (6.4 ± 4.5 vs. 8.5 ± 5.2) compared with follitropin alfa.

The average fertilization rate in women with oocytes retrieved was approximately 63% in both treatment groups (Table 2). The proportion of women with embryo transfer was 85.2% (322/378) in the follitropin delta group and 81.4% (310/381) in the follitropin alfa group (Fig. 3). The mean number of embryos (7.3 ± 4.7 vs. 8.8 ± 5.5) and the number of embryos with good quality (4.4 ± 3.8 vs. 5.3 ± 4.2) on day 3 after oocyte retrieval were both lower in the follitropin delta than that in the follitropin alfa group. Similar numbers (7.4 ± 4.9 vs. 9.7 ± 5.7 and 4.6 ± 3.9 vs. 5.9 ± 4.5) were observed in the two stimulation groups in women with AMH ≥ 15 pmol/l (Table 3). However, there were no significant difference between two groups in women with AMH < 15 pmol/l.

### OHSS incidence

There were no cycle cancellations due to excessive ovarian response. The incidence of preventive interventions for early OHSS by GnRH agonist triggering (1.6% vs. 4.2%) and the incidence of early OHSS and/or preventive interventions (6.1% vs. 11.0%)) was lower in the follitropin delta group than in the follitropin alfa group (Table 2). However, the overall incidence of OHSS and the incidence of early or late OHSS was not statistically different between the two treatment groups.

In women with AMH < 15 pmol/L, the incidence of early OHSS and the incidence of preventive intervention for early OHSS was similar between the two groups (Table 3), whereas in women with AMH ≥ 15 pmol/l, the incidence of early OHSS (5.0% vs. 9.5%) and the incidence of preventive interventions for early OHSS (2.0% vs. 5.1%) were both lower in the follitropin delta group than those in the follitropin alfa group.

### Dose equivalence factor

**Fig. 4:**
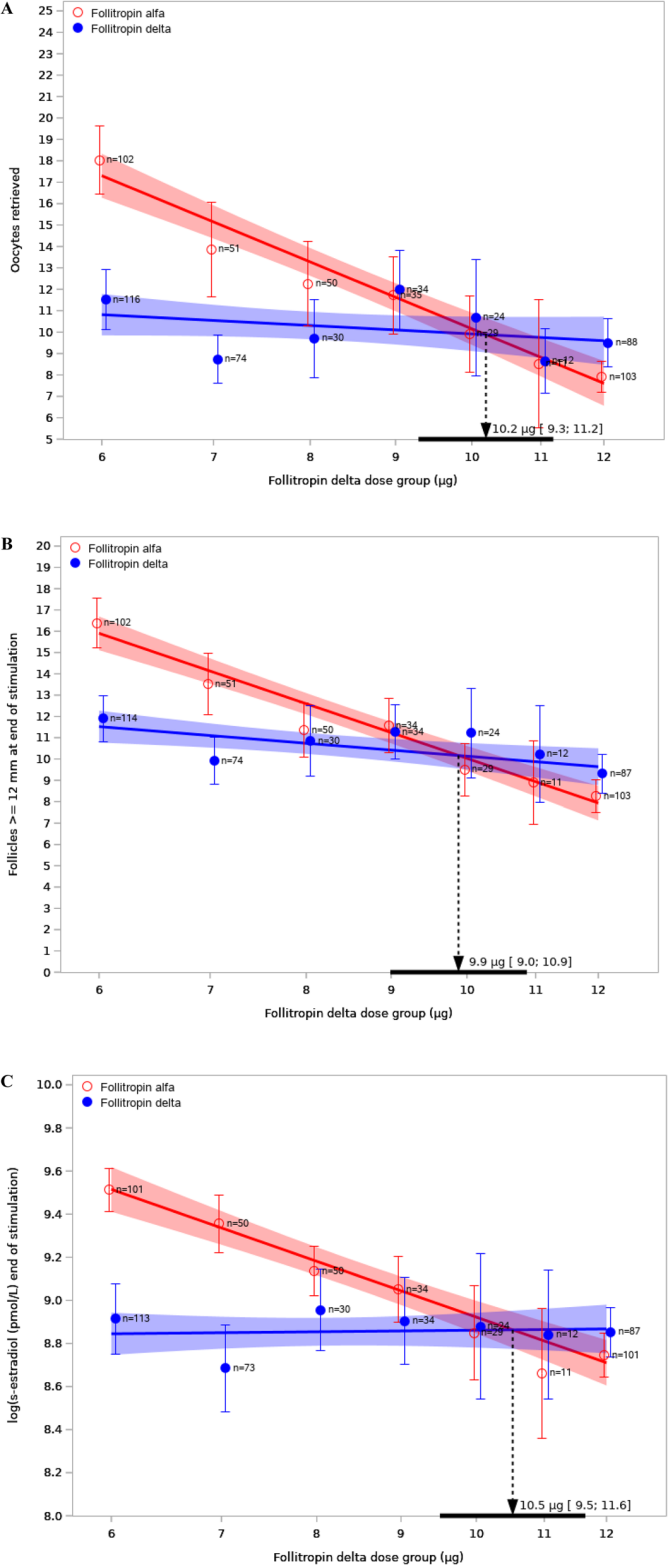
Analysis of dose equivalence. Note: **A** Number of oocytes retrieved for follitropin delta and 150 IU/day follitropin alfa in the study (*n* = 759); **B** Number of follicles ≥ 12 mm at end of stimulation for follitropin delta and 150 IU/day follitropin alfa in the study (*n* = 759); **C** Log of serum oestradiol concentrations at the end of stimulation for follitropin delta and 150 IU/day follitropin alfa in the study (*n* = 759). Estimated means (circles) with 95% confidence intervals (95% CI) and number of patients for the subgroups based on the dose of follitropin delta corresponding to the patients’ AMH concentrations and body weight. The intersection of the blue and red regression lines indicates the dose of follitropin delta estimated to give the same response as 150 IU of follitropin alfa. The estimate dose equivalence factor and its 95% CI are indicated by the arrow and the solid horizontal black line on the x-axis

The dose equivalence factor between follitropin delta and follitropin alfa are illustrated in Fig. 4A-C. The intersection of the blue (follitropin delta) and red (follitropin alfa) regression lines (fitted to the individual data points) is the follitropin delta dose estimated to give the same ovarian response as treatment with follitropin alfa at a starting dose of 150 IU/ day. The follitropin delta dose providing similar number of oocytes retrieved as 150 IU of follitropin alfa is estimated to 10.2 μg (95% CI: 9.3–11.2) (Fig. 4A).

Similarly, the follitropin delta dose providing similar number of follicles with a diameter ≥ 12 mm (Fig. 4B) and similar oestradiol concentrations (Fig. 4C) on the last stimulation day as 150 IU of follitropin alfa were 9.9 μg (95% CI: 9.0–10.9) and 10.5 μg (95% CI: 9.5–11.6), respectively.

## Discussion

This study reports on a preplanned subgroup analysis in Chinese IVF/ICSI patients participating in the GRAPE study [14] which was adequately powered for the primary endpoint ongoing pregnancy. This study compared ovarian stimulation with individualized dosing of follitropin delta, based on pretreatment AMH concentrations and body weight, to follitropin alfa dosed according to label. Non-inferiority of follitropin delta compared to follitropin alfa was confirmed among women recruited from mainland China only, which was consistent with the overall population in the Pan-Asian study [14]. Moreover, the live birth rates of 31.0% for individualized dosing of follitropin delta and 25.5% for conventional dosing of follitropin alfa confirmed the favorable ongoing pregnancy rates in Chinese IVF/ICSI patients. In comparison to previous comparative trials of follitropin delta in European [1] and Japanese [13], Chinese patients were younger, with a lower body weight and higher serum AMH levels thus received in this study more frequently a lower dose of follitropin delta. The proportion of potential high responders (subjects with AMH ≥ 15 pmol/l) was almost 80% in the Chinese subgroup, whereas the corresponding numbers were 55% in the European trial and 59% in the Japanese trial. This, together with the lower body weight resulted in a lower mean follitropin delta dose administrated in this trial, namely 76.7 μg in Chinese patients compared to 90.0 μg in European and 83.5 μg in Japanese patients. Hence, the overall results in the Chinese population are closer to those of potential high responders in the European trial who required only 67.4 µg follitropin delta [17].

Overall, due to individualized dosing in the follitropin delta group, patients in this trial developed significantly fewer follicles at the end of stimulation with lower serum oestradiol, progesterone, inhibin A and inhibin B levels in the follitropin delta group than that in the follitropin alfa group. This overall lower ovarian response was mainly due to potential high responders (as indicated by serum AMH ≥ 15 pmol/L), who had a lower average oocyte yield i.e. 10.5 for follitropin delta and 13.9 for follitropin alfa, indicating that stimulation with follitropin delta also normalizes ovarian response in Chinese high responders. In contrast, in potential low responders (as indicated by serum AMH < 15 pmol/L), more oocytes were retrieved in the follitropin delta group than in the follitropin alfa group (mean 9.2 vs 7.7). These results are in agreement with previous controlled trials of follitropin delta in Europe and Asia [1, 14] although in the current trial relatively more potential high responders were recruited. Regardless the population, there were no significant differences between groups in terms of percentage of women with embryo transfer, and the ongoing pregnancy rates were unaffected, indicating no difference in the quality of embryos transferred.

Thanks to the normalized ovarian response of potential high responders treated with follitropin delta, a lower incidence of early OHSS and/or incidence of preventive interventions was observed for women with follitropin delta compared to women treated with follitropin alfa. The improved safety outcome in this trial is in line with the analysis of the ESTHER-1 and ESTHER-2 study [19] showing a 50% reduction in incidence of moderate/severe OHSS with individualized follitropin delta compared with conventional dosing approach. For potential high responders, applying algorithm-based dosing of follitropin delta determines the dose of follitropin delta by incorporating a women’s serum AMH and body weight can mitigate the risk of OHSS due to ovarian stimulation. Although GnRH agonist triggering and freezing of all embryos lowers the risk of OHSS, patients have to wait until at least one more cycle for embryo transfer which delays their time to pregnancy [3].

The dose equivalence factor was assessed in this study to determine which dose of follitropin delta provides in Chinese women similar ovarian response as 150 IU of follitropin alfa. It was estimated that a daily follitropin delta dose of ≈10 µg would provide the same ovarian response as a starting dose of 150 IU/d of follitropin alfa (11 µg). This dose equivalence factor in Chinese women is consistent with the previous calculated factor for European and other western women [16] and confirms that one µg follitropin delta is more potent than one µg of follitropin alfa. Understanding the dose equivalence factor may help clinicians who are accustomed to IU doses to understand the anticipated ovarian response to different doses of follitropin delta and avoid dosing misunderstandings/errors.

In this trial the average total dose was 76.7 µg in the follitropin delta group and 1505 IU (110.4 µg) in the follitropin alfa group. Applying the dose equivalence factor, a follitropin delta dose of 76.7 µg was estimated to provide the same ovarian response as 1150.5 IU follitropin alfa. In this trial, the follitropin alfa dose was on average 354.5 IU more per patient than what was needed to get the same ovarian response as subjects treated with individualized follitropin delta. The difference in dose between the two groups was larger in the current study than in previous comparative studies, as the Chinese population included relatively more women with AMH ≥ 15 pmol/L and a lower body weight [1, 13].

China has the largest population in the world. Currently, ART services are not covered by any medical insurance system at national level in China. Although the development of ART in mainland China has presented a rapid growth, imbalance of ART service still exists geographically [20, 21]. In China, patients tend to seek public hospitals in the eastern big cities for ART service far away from their domestic region [22]. Regardless travel distance, more patient-friendly and safer IVF treatments with less complications and visits to the clinic are the pursuit for all infertile patients. Individualized fixed dose treatment regimens are a next step in the right direction as evidenced by the outcome of this analysis in the Chinese IVF population.

## Strengths and limitations

### Advantages

This is the first efficacy and safety analysis of the individualized follitropin delta treatment compared to conventional follitropin alfa treatment in Chinese IVF patients. The data presented confirmed the benefits of follitropin delta dosing based on AMH and body weight, regardless ethnicity, resulting in a balanced efficacy and safety outcome with decreased risk of OHSS and reduced gonadotropin use.

### Limitations

This study concerns a subgroup analysis of GRAPE study limited to Chinese patients, thus excluding patients outside mainland China. In addition, the live births were collected as follow-up data and the dose equivalence factor was a retrospective analysis. The cost-effectiveness of follitropin delta versus follitropin alfa in China should be further analyzed to provide guidance for clinicians and patients.

## Conclusions

Individualized follitropin delta dosing was shown to be safer than conventional follitropin alfa in Chinese patients, as it decreased the incidence of early OHSS and/or preventive interventions whereas much lower amounts of FSH were required to reach the same triggering criteria.

## Supplementary Information

**Additional file 1:** **Additional Figure 1.** Study design and study timeline. AE, adverse event; OHSS, ovarian hyperstimulation syndrome; GnRH, gonadotropin-releasing hormone

## Data Availability

The dataset used and analysed underlying this article are available from the corresponding author on reasonable request.

## Abbreviations

ART: Assisted reproductive technology
COS: Controlled ovarian stimulation
AMH: Anti-Müllerian hormone
IVF: In vitro fertilization
AFC: Antral follicle count
OHSS: Ovarian hyperstimulation syndrome
FSH: Follicle stimulating hormone
ICSI: Intracytoplasmic sperm injection
hCG: Human chorionic gonadotrophin
GnRH: Gonadotropin-releasing hormone
CI: Confidence interval
OR: Odds ratio
SD: Standard deviation
LH: Luteinising hormone
AE: Adverse event
