# Supplementary figures and images for: Comparative clinical outcome following individualized follitropin delta dosing in Chinese women undergoing ovarian stimulation for in vitro fertilization /intracytoplasmic sperm injection
Source: Reprod Biol Endocrinol. 2022 Oct 4;20:147. doi: 10.1186/s12958-022-01016-y (PMC9531501; doi:10.1186/s12958-022-01016-y)

**Additional Figure 1.**


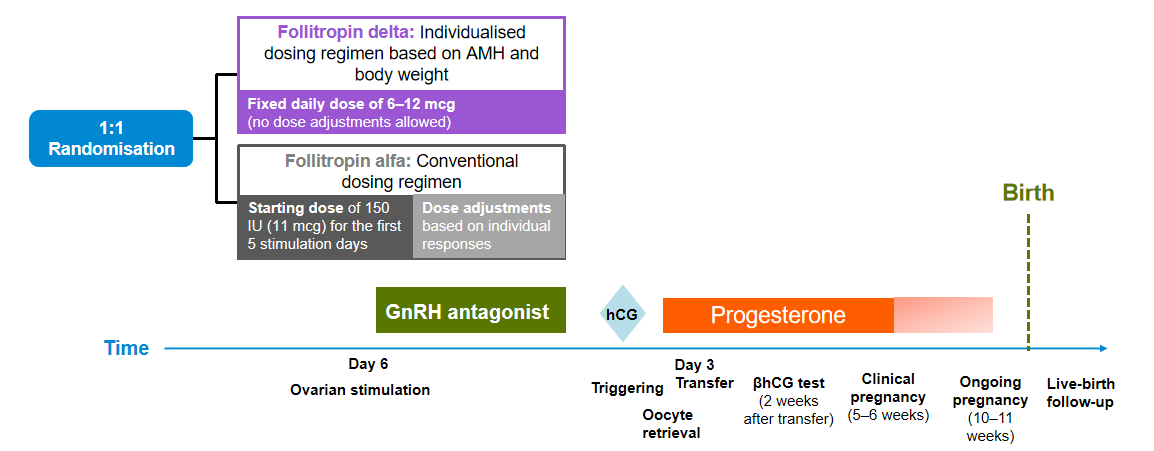

Supplement: Supplementary file 1 — Additional file 1: Additional Figure 1. Study design and study timeline. AE, adverse event; OHSS, ovarian hyperstimulation syndrome; GnRH, gonadotropin-releasing hormone [file 12958_2022_1016_MOESM1_ESM.docx]
